# Paeonol Ameliorates Diabetic Renal Fibrosis Through Promoting the Activation of the Nrf2/ARE Pathway via Up-Regulating Sirt1

**DOI:** 10.3389/fphar.2018.00512

**Published:** 2018-05-18

**Authors:** Lei Zhang, Zhiquan Chen, Wenyan Gong, Yezi Zou, Futian Xu, Lihao Chen, Heqing Huang

**Affiliations:** ^1^School of Pharmaceutical Sciences, Guangzhou University of Chinese Medicine, Guangzhou, China; ^2^Laboratory of Pharmacology and Toxicology, School of Pharmaceutical Sciences, Sun Yat-sen University, Guangzhou, China

**Keywords:** paeonol, oxidative stress, diabetic nephropathy, Sirt1, Nrf2/ARE pathway

## Abstract

Diabetic nephropathy (DN) is rapidly becoming the leading cause of end-stage renal disease worldwide and a major cause of morbidity and mortality in patients of diabetes. The main pathological change of DN is renal fibrosis. Paeonol (PA), a single phenolic compound extracted from the root bark of Cortex Moutan, has been demonstrated to have many potential pharmacological activities. However, the effects of PA on DN have not been fully elucidated. In this study, high glucose (HG)-treated glomerular mesangial cells (GMCs) and streptozotocin (STZ)-induced diabetic mice were analyzed in exploring the potential mechanisms of PA on DN. Results *in vitro* showed that: (1) PA inhibited HG-induced fibronectin (FN) and ICAM-1 overexpressions; (2) PA exerted renoprotective effect through activating the Nrf2/ARE pathway; (3) Sirt1 mediated the effects of PA on the activation of Nrf2/ARE pathway. What is more, in accordance with the *in vitro* results, significant elevated levels of Sirt1, Nrf2 and downstream proteins related to Nrf2 were observed in the kidneys of PA treatment group compared with model group. Taken together, our study shows that PA delays the progression of diabetic renal fibrosis, and the underlying mechanism is probably associated with regulating the Nrf2 pathway. The effect of PA on Nrf2 is at least partially dependent on Sirt1 activation.

## Introduction

Diabetic nephropathy is not only one of the major microvascular complications of diabetes, but also the leading cause of end-stage renal disease. The main pathological change of DN is renal fibrosis, including glomerulosclerosis and tubulointerstitial fibrosis. Constituting the central position in the glomerulus, GMCs are increasingly recognized to be primary contributors to fibrotic lesion in kidneys. Over-proliferation of GMCs, followed by excessive accumulation of ECM proteins like FN and over-secretion of inflammatory cytokines like ICAM-1, trigger the thickening of GBM and expansion of mesangial area, accelerating the progression of renal fibrosis to develop into DN at last (Kanwar et al., 2011; Kolset et al., 2012).

The etiology of DN is multifactorial and includes changes of renal hemodynamics, polyol pathway dysfunction, oxidative stress and activation of inflammatory pathway (Evans et al., 2002; Dronavalli et al., 2008). Accumulating evidence suggests that oxidative stress, which reflects an imbalance between the systemic manifestation of ROS and a biological system’s ability to eliminate the reactive intermediate or to repair the body damage, acts as the common denominator linking altered metabolic pathways in the kidneys related with DN (Kanwar et al., 2008). Specifically, under diabetes, ROS function as the mediator and signal amplifier to activate downstream signal transduction pathways, including p38MAPK, ERK1/2, and NF-κB, etc. These factors interact with one another and facilitate inflammatory and fibrotic processes, ultimately leading to glomerulosclerosis (Brownlee, 2001; Ha and Lee, 2005; Tan et al., 2007). Therefore, appropriate regulation of oxidative stress responses is imperative during clinical treatment for DN.

The Nrf2/antioxidant responsive element (Nrf2/ARE) signaling pathway is one of the most important regulators of cellular antioxidant responses (Jiang et al., 2010; Zheng et al., 2011; Arellano-Buendia et al., 2016). Under normal conditions, Nrf2 is maintained in the cytoplasm as an inactive complex bound to the repressor protein Kelch-like ECH-associated protein 1 (Keap1). When suffering oxidative and electrophilic stimulation, Nrf2 dissociates from Keap1 and then translocates to the nucleus where it binds to the ARE to regulate the transcription of numerous antioxidant and cytoprotective genes, such as HO-1 and SOD1 (Kobayashi and Yamamoto, 2006; Kensler et al., 2007; Kim and Vaziri, 2010). It protects cells against oxidative stress insult by decreasing overproduction of FN and ICAM-1, thus inhibiting the oxidative stress-induced initiation and progression of diabetic renal fibrosis (Li et al., 2012). Hence, it was of therapeutic significance to activate the Nrf2 pathway in the anti-oxidant therapy for DN.

Sirt1, as a protein deacetylase, is a critical enzyme for prevention of cellular oxidative stress (Chong and Maiese, 2008). Previous studies have reported that Sirt1 participates in the regulation of DNA repair and recombination, caloric restriction, lifespan, inflammation and cancer (Rahman and Islam, 2011; Stunkel and Campbell, 2011; Zhang et al., 2011; Canto and Auwerx, 2012; Martinez-Redondo and Vaquero, 2013). Recently, increasing numbers of studies have focused on the involvement of Sirt1 in kidney diseases and Sirt1 has become a promising target for DN treatment (Yeung et al., 2004; Kume et al., 2006, 2010). We and others have showed the beneficial advantages of Sirt1 on the treatments for various kidney diseases through regulating metabolic disorder, inflammatory and anti-oxidative responses (Kume et al., 2010; Fan et al., 2011; Huang et al., 2013).

Paeonol, a simple phenolic compound that can be extracted from Moutan Cortex and *Cynanchum paniculatum*, has a vast array of biological and pharmacological activities regarding anti-inflammation, anti-tumor, anti-hypertension, and anti-oxidation, etc. (Sun et al., 2008; Chen et al., 2012; Zhang et al., 2013; Gong et al., 2017). Recent studies reveal that PA treatment reduces blood glucose level in STZ-induced diabetic rats (Liu et al., 2013) and decreases content of FN and TGF-β1 in the supernatant of HG-cultured mesangial cells (Sun et al., 2015) separately, which indicate the potential renoprotective effect of PA. However, the molecular mechanism by which PA exerts renoprotective activity remains unclear.

Given that anti-oxidant treatment is beneficial for DN patients, there is a pressing need to better understand whether the anti-oxidant activity of PA contributes to its renoprotective activity during DN. In this study, we evaluated the effects of PA on diabetic renal fibrosis in HG-induced GMCs and STZ-induced diabetic mice and further assessed the underlying mechanisms with specific focus on the Nrf2 pathway. Data showed that the protective effect of PA on DN is through activating the Nrf2/ARE pathway. Underlying mechanism for the activation is, at least partially, through up-regulating Sirt1.

## Materials and Methods

### Chemical Reagents and Antibodies

DMEM and fetal bovine serum (FBS) were purchased from Life Technologies (Grand Island, NY, United States). PA used for cell treatment was purchased from National Institutes for Food and Drug Control (Guangzhou, China). PA used in animal trial was from Zelang (purity > 98.0%, HPLC; Nanjing, China). Metformin Hydrochloride Tablets used in animal experiments were purchased from Bristol-Myers Squibb Company (Shanghai, China). DMSO and STZ were purchased from Sigma-Aldrich (St. Louis, MO, United States). Primary antibodies against FN (catalog: sc-18825) and ICAM-1 (catalog: sc-1511) were from Santa Cruz Biotechnology (Dallas, TX, United States); Antibodies against Nrf2 (catalog: 16396-1-AP) and SOD-1 (catalog: 10269-1-AP) were purchased from Proteintech Group (Wuhan, China); Sirt1 (catalog: BS6494) was from Bioworld Technology (St. Paul, MN, United States); HO-1 (catalog: A1346) was from Abclonal Technology (Baltimore Avenue, United States); Lamin B1 (catalog: ab-133741) was from Abcam (Cambridge, MA, United States); Anti-rabbit and anti-goat secondary antibodies were purchased from Beyotime (Haimen, China); Alexa Fluor^®^ 488 goat anti-rabbit IgG (catalog: A-11008) was purchased from Molecular Probes (Eugene, OR, United States).

### Cell Culture and PA Treatment

Rat GMCs were isolated from kidney cortex according to the protocol described previously (Geoffroy et al., 2004). They were grown in Dulbecco’s modified Eagle’s medium supplemented with 10% fetal bovine serum at 37°C in a humidified incubator containing 5% CO_2_. GMCs were sub-cultured at a ratio of 1:6 when the confluence reached 90%. Cells from passages 3 to 12 were used for study. All cultured GMCs were starved overnight before treatments. PA was solubilized in DMSO at 20 mg/mL as mother liquor and stored at -80°C for long preservation. At appropriate subconfluence, cells were serum-starved overnight followed by the presence or absence of PA at varying concentrations for 2 h, and then they were co-treated with HG and PA for indicated time. In this study, 5.6 mM glucose and 30 mM glucose were used as control group and model group separately.

### MTT Assay

In this study, the 3- (4,5-dimethylthiazol-2-yl) -2,5-diphenyl tetrazolium bromide (MTT) assay was used to evaluate the effects of PA on the cell viability of GMCs. GMCs were seeded in the 96-well plate at the appropriate density. After adherence, GMCs were starved for 12 h in serum-free DMEM medium, and then treated with different concentrations of PA for another 24 h. 20 μL MTT (0.5 mg/mL) was added to each well and incubated continuously for 2 h at 37°C. The cells were then treated with 200 μL DMSO. The best measurement degree of 570 nm was selected to detect the absorbance (570 nm) of each well was measured by spectrophotometry (BioTek, United States).

### Western Blotting (WB)

Sirt1, Nrf2, HO-1, SOD1, FN, and ICAM-1 expression levels were measured using western blot in accordance with the previously reported method (Huang et al., 2017). Lamin B1 and α-tubulin were used as loading controls.

### Small Interfering RNA (siRNA)

The specific siRNA sequences, including Sirt1-siRNA, Nrf2-siRNA and the negative control, were synthesized by Sangon Biotech (Shanghai, China). The sequences of Sirt1-siRNA were as follows: sense: 5′-CCAGUAGCACUAAUUCCAATT-3′, antisense: 5′-UUGGAAUUAGUGCUACUGGTT-3′. The sequences of Nrf2-siRNA were as below: sense: 5′-GAGGAUGGGAAACCUUACUTT-3′, antisense: 5′-AGUAAGGUUUCCCAUCCUCTT-3′. Appropriate siRNA was transiently transfected into GMCs with lipofectamine^®^ RNAiMAX (Invitrogen, United States). After 48 h incubation, the transfection medium was replaced with fresh serum-free DMEM for another 12 h. After various treatments, the cells were harvested for WB.

### Immunofluorescence

Glomerular mesangial cells were seeded at a proper density on the coverslips in the wells of 24-well plate and then treated with the corresponding treatments when 20% of confluence. After rinsed with cold PBS for three times, cells were fixed with paraformaldehyde solution (final concentration of 4%) for 20 min and permeabilized with 1% Triton-100 for 5 min. Blocked with 10% goat serum for 1 h, cells were incubated with the corresponding antibodies on a shaking table at 4°C overnight or room temperature for 2 h. The cells were washed again, incubated with the corresponding second fluorescent antibodies for 1 h in the dark, and then counterstained with DAPI for 10 min. The fluorescent levels were detected by laser confocal (Carl Zeiss, Germany).

### Analysis of Intracellular Superoxide and H_2_O_2_

Intracellular superoxide and H_2_O_2_ were detected to reflect ROS levels. The superoxide level was measured by using fluorescent probe dihydroethidium (DHE). After various treatments, cells were rinsed with fresh medium for three times, and then supplemented with DHE (10 μM) in serum-free DMEM medium at 37°C for 30 min. The fluorescence intensity was detected by high content screening (Thermo Fisher, United States). As for H_2_O_2_, we conducted the assays according to the manual of the kit (Beyotime, China) strictly. Briefly, cells were collected from 6-well plate using lysis buffer provided by the kit. The cells were completely lysed on ice followed by centrifugation at 4°C, 1,2000 *g* for 5 min 50 μL supernatant was mixed with 100 μL detection reagent, and then the mixture was placed at room temperature for 30 min. The absorbance at 560 nm was measured. By substitution of the values which obtained from spectrometer at 560 nm into the standard curve premade, the concentrations of H_2_O_2_ in each group were calculated.

### Electrophoretic Mobility Shift Assay (EMSA)

Electrophoretic mobility shift assay (EMSA) was used to detect the DNA-binding activity of Nrf2. Firstly, nuclear proteins were extracted from cultured cells according to the protocol (Active Motif, United States) as previously described (Chen Q. et al., 2017). The sequences of biotin labeled oligonucleotide probe for ARE were as follows: 5′-ACTGAGGGTGACTCAGCAAAATC-3′, 3′-TGACTCCCACTGAGTCGTTTTAG-5′ (Beyotime, China). 5 μg nuclear samples were mixed with other components [50 ng/μL poly (dI-dC), 0.05% NP-40, 5 mM MgCl_2_ and 2.5% glycerol], incubated for 10 min and then mingled with 2 μL ARE probe for 20 min. The reaction mixture was loaded on the 6% non-denaturing polyacrylamide gel to separate and then transferred onto the NC membrane. Membrane was exposed to ultraviolet light for 15 min, and then incubated for 1 h at room temperature. 33.3 μL stabilized streptavidin-horseradish peroxidase conjugate was added into blocking buffer to yield a total volume of 10 mL. The brands were visualized with enhanced chemiluminescence.

### Animals

Male C57/BL6 mice (*n* = 40, 20 ± 2 g) were purchased from Laboratory Animal Center, Sun Yat-sen University, Guangzhou, China (Animal Quality Certificate Number: 44007200039420). Animal experiments were in accordance with the National Institutes of Health guide for the care and use of Laboratory animals.

Before the experiment, mice were adapted to the environment for 1 week with high-fat feed. The diabetic models were generated by intraperitoneal injection of STZ (50 mg/kg) for 5 days. Mice with FBG value higher than 11.1 mM were identified as successful models. The mice were randomized to four treatment groups: (1) control group (*n* = 8), (2) diabetic group (*n* = 8), (3) PA treatment group (*n* = 8, 150 mg/kg, six times/week), (4) metformin treatment group (*n* = 8, 195 mg/kg; six times/week). The dose of PA was selected based on previously published data on the anti-diabetic effect of PA (Lau et al., 2007). Animals were kept fasted overnight prior to the end of 8-week experiment, then they were sacrificed to obtain serum and kidney samples.

### Biochemical Analysis

Mice were housed in metabolic cages with free access to sterile water to collect 24 h urine before the end of the experiment. FBG was measured by One-Touch glucometer. BUN, Cr and 24 h UP were measured by using the manufacturer’s protocols from Nanjing Jiancheng Biology Engineering Institute (China).

### Statistics

One-way ANOVA analysis was performed by GraphPad Prism 5.0 and the results were expressed as mean ± SEM. A *p*-value < 0.05 was defined statistically significant.

## Results

### PA Decreased HG-Induced ROS Production and Expressions of FN and ICAM-1

**FIGURE 1:**
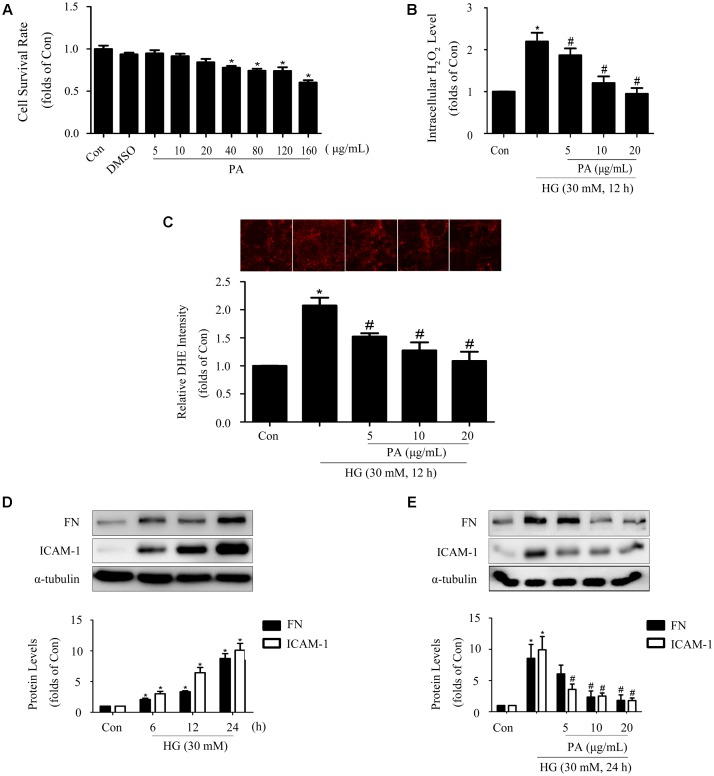
Paeonol (PA) decreased HG-induced ROS production and expressions of FN and ICAM-1. **(A)** Cell survival analysis through MTT assay of GMCs treated with different concentrations of PA for 24 h. **(B)** Quantification of H_2_O_2_ in GMCs treated with 30 mM HG for 12 h. **(C)** Analysis of DHE fluorescence intensity. **(D)** Western blot analysis of FN and ICAM-1 in GMCs treated with 30 mM HG at different time. **(E)** Western blot analysis of FN and ICAM-1 in GMCs treated with 30 mM HG and/or PA for 24 h. The above experiments were performed at least three times with similar results. ^∗^*P* < 0.05 vs. Con, ^#^*P* < 0.05 vs. HG. See Supplementary Figure 1 for uncropped scans of the western blot.

Data from MTT showed appropriate concentrations of PA with no significant cytotoxicity to GMCs. Here, 5, 10, and 20 μg/mL PA were used in the subsequent studies (**Figure 1A**). It has been reported that overproduction of ROS incurs oxidative stress which contributes to the pathogenesis of diabetic complications (Manucha et al., 2015), including DN. As confirmed by our previous study (Chen Z. et al., 2017), treatment with HG (12 h) induced notable increases in ROS generations in GMCs, and therefore the 12 h time point was adopted to evaluate the effect of PA on ROS levels. The results showed that H_2_O_2_ and superoxide levels increased by twofold in GMCs exposed to HG for 12 h, whereas these increments were both reduced by PA treatment (**Figures 1B,C**). Moreover, 12 h of HG treatment also triggered significant up-regulations of inflammatory fibrotic factors, such as FN and ICAM-1 which increased in time-dependent manners within 24 h (**Figure 1D**). However, PA suppressed these up-regulations induced by HG 24 h (**Figure 1E**). All data above indicated that PA was a potent compound to inhibit the development of diabetic renal fibrosis, and the underlying mechanism might be correlated with resisting oxidative stress.

### Effects of PA on the Nrf2 Pathway in GMCs Treated With Short-Time HG

**FIGURE 2:**
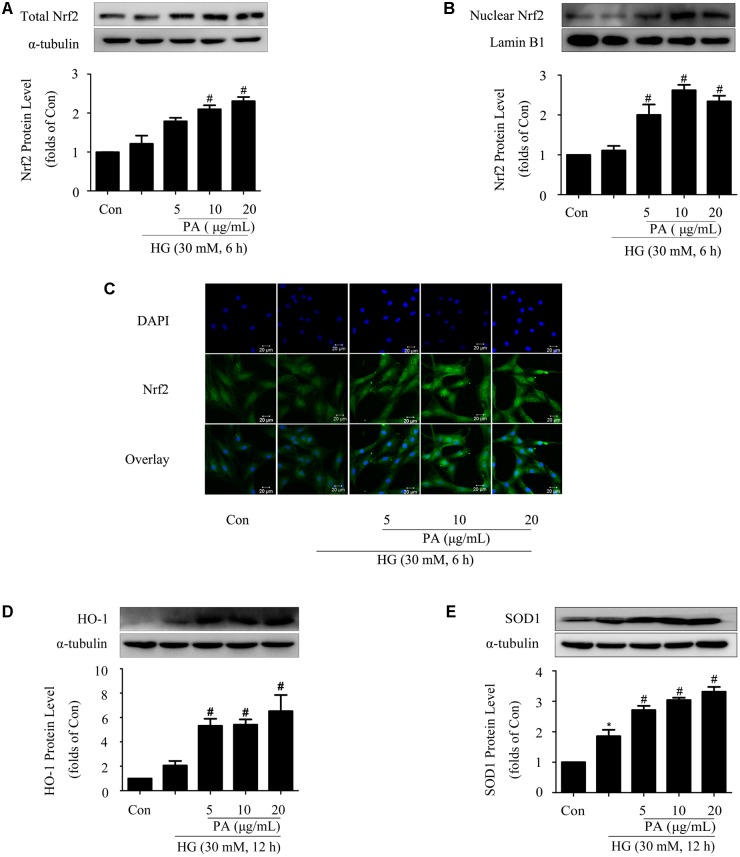
Effects of PA on the Nrf2 pathway in GMCs treated with short-time HG. The GMCs were stimulated with high glucose for another 6 or 12 h after pretreatment with 5, 10, and 20 μg/mL PA for 2 h. **(A,B)** Western blot analysis of total Nrf2 and nuclear Nrf2 in GMCs treated with 30 mM HG and/or PA for 6 h. **(C)** Representative immunofluorescence images of Nrf2 (green) translocation into the nuclei (labeled with DAPI dye) of GMCs untreated or treated with PA. **(D,E)** Western blot analysis of HO-1 and SOD1 in GMCs treated with 30 mM HG and/or PA for 12 h. The above experiments were performed at least three times with similar results. ^∗^*P* < 0.05 vs. Con, ^#^*P* < 0.05 vs. HG. See Supplementary Figure 2 for uncropped scans of the western blot.

Considering that Nrf2/ARE is one of the classic intracellular antioxidant signaling pathways, western blotting was performed to investigate the effects of PA on the Nrf2 pathway. As we have shown in previous study, the Nrf2 pathway was adaptively activated first (within 6 h) and then declined (Huang et al., 2013). Based on these results, we determined to investigate the effects of PA on Nrf2 activation at short- and long-time points of HG treatment. Our results showed that HG treatment for 6 h had no obvious effect on total Nrf2 expression and Nrf2 nuclear translocation. PA treatment increased total Nrf2 expression and promoted Nrf2 nuclear translocation (**Figures 2A,B**). Confocal analysis reconfirmed the increased amount of Nrf2-positive nuclei induced by PA (**Figure 2C**).

SOD1 expression was adaptively up-regulated (**Figure 2E**) during the observation time (12 h), while the increase of HO-1 level was not statistically significant (**Figure 2D**) after 12 h of HG treatment. Notably, PA treatment further enhanced the expressions of HO-1 and SOD1 in a dose-dependent manner (**Figures 2D,E**).

### Effects of PA on the Nrf2 Pathway in GMCs Treated With Long-Time HG

**FIGURE 3:**
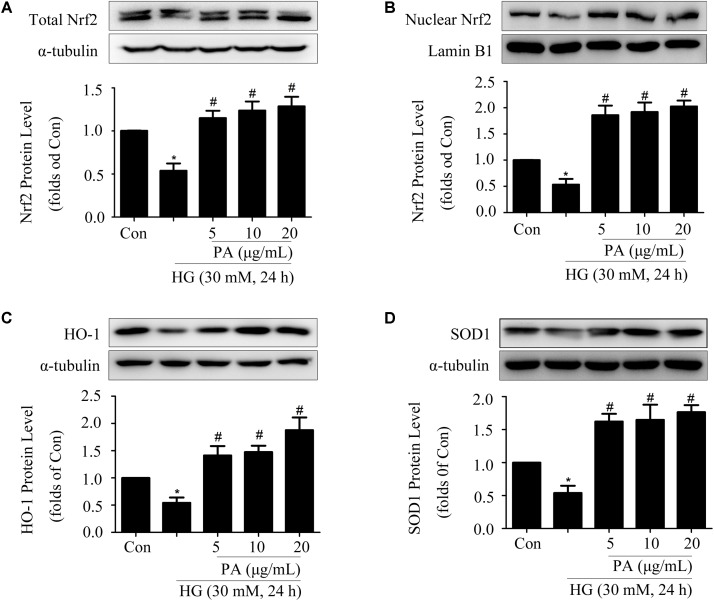
Effects of PA on the Nrf2 pathway in GMCs treated with long-time HG. All PA pretreatments were performed for 2 h before 30 mM HG treatment for another 24 h. **(A,B)** Western blot analysis of tota1 and nuclear Nrf2 in GMCs treated with HG and/or PA for 24 h. **(C,D)** Western blot analysis of HO-1 and SOD1 in GMCs with or without PA treatment. The above experiments were performed at least three times with similar results. ^∗^*P* < 0.05 vs. Con, ^#^*P* < 0.05 vs. HG. See Supplementary Figure 3 for uncropped scans of the western blot.

Since PA played a positive role in the activation of Nrf2/ARE pathway within a short time (6–12 h), then we assessed the effects of PA on the Nrf2 pathway under long-time HG treatment (24 h) conditions. HG treatment for 24 h significantly decreased the expression and nuclear translocation of Nrf2 in GMCs, but PA treatment reversed the decline of total Nrf2 expression and further promoted the nuclear content of Nrf2 (**Figures 3A,B**). In response to long-time HG stimulus, HO-1 and SOD1 expressions showed sharp declines in GMCs, but these decreases were likewise reversed by PA (**Figures 3C,D**).

### Activation of the Nrf2 Pathway Was Required for PA to Reduce FN and ICAM-1 Expressions in HG-Induced GMCs

**FIGURE 4:**
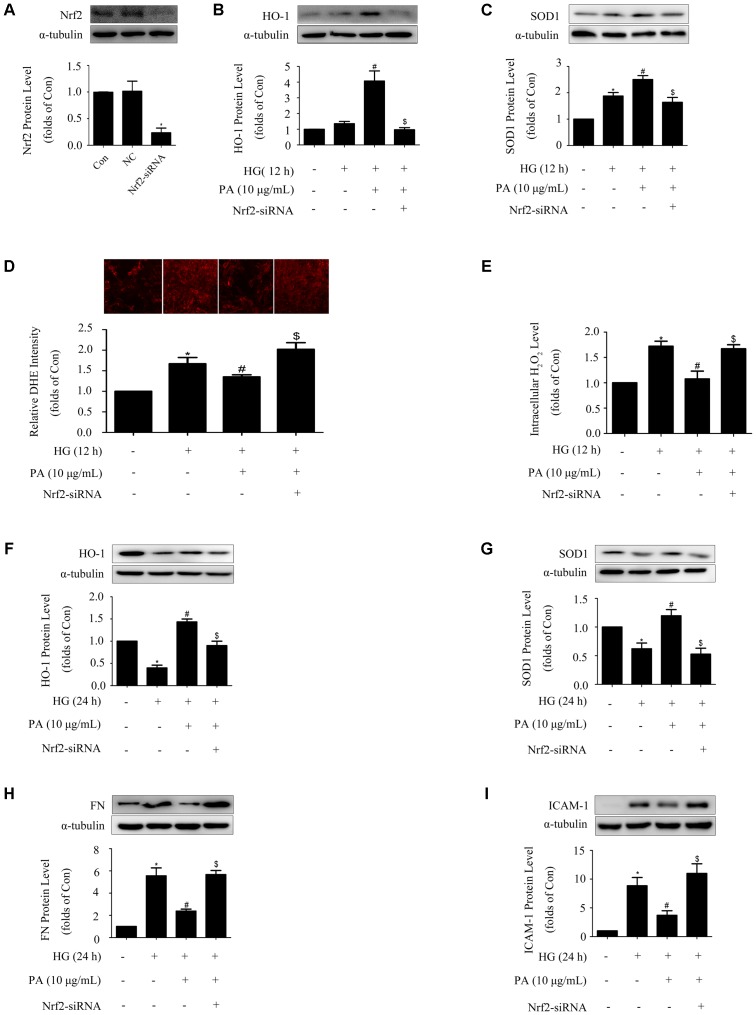
Activation of the Nrf2 pathway was required for PA to reduce FN and ICAM-1 expressions in HG-induced GMCs. GMCs were transiently transfected with Nrf2-siRNA, after which cells were treated with HG for 12 or 24 h. **(A)** Confirmation of Nrf2-siRNA silencing efficiency. **(B,C)** Effects of Nrf2-siRNA on HO-1 and SOD1 expressions in GMCs under HG (12 h) conditions. **(D)** Quantitative analysis of superoxide production by DHE staining. **(E)** Quantification of H_2_O_2_ in GMCs under Nrf2 knockdown conditions. **(F,G)** Effects of depletion of Nrf2 on HO-1 and SOD1 expressions in GMCs under HG (24 h) conditions. **(H,I)** FN and ICAM-1 expressions under Nrf2 knockdown conditions. The above experiments were performed at least three times with similar results. ^∗^*P* < 0.05 vs. Con, ^#^*P* < 0.05 vs. HG, ^$^*P* < 0.05 vs. HG + PA. See Supplementary Figure 4 for uncropped scans of the western blot.

To further investigated the mechanism by which PA inhibited expressions of FN and ICAM-1, knockdown of Nrf2 was performed and results of WB showed that the protein level of Nrf2 was efficiently knocked down by 80% (**Figure 4A**). Concomitant with knockdown of Nrf2, PA treatment no longer up-regulated HO-1 and SOD1 expressions under both short-time and long-time HG states (**Figures 4B,C,F,G**). Crucially, Nrf2-siRNA abrogated the reductions of superoxide and H_2_O_2_ induced by PA (**Figures 4D,E**), and ultimately reversed the inhibitory effects of PA on expressions of FN and ICAM-1 (**Figures 4H,I**). These results indicated that the Nrf2/ARE pathway was involved in the regulatory processes of PA on expressions of FN and ICAM-1.

### PA Up-Regulated Expression and Nuclear Accumulation of Sirt1 in HG-Induced GMCs

**FIGURE 5:**
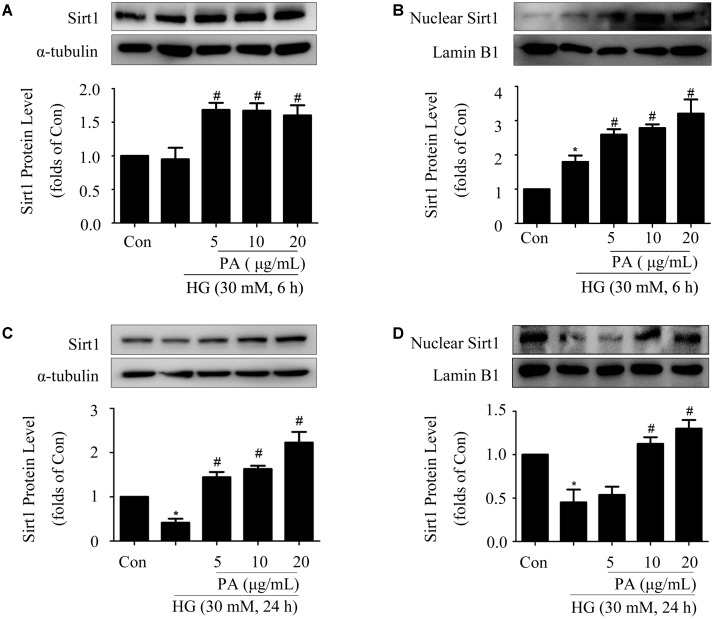
Paeonol up-regulated expression and nuclear accumulation of Sirt1 in HG-induced GMCs. **(A,B)** Western blot analysis of total Sirt1 and nuclear Sirt1 in GMCs treated with 30 mM HG and/or PA for 6 h. **(C,D)** Western blot analysis of total Sirt1 and nuclear Sirt1 in GMCs treated with HG and/or PA for 24 h. The above experiments were performed at least three times with similar results. ^∗^*P* < 0.05 vs. Con, ^#^*P* < 0.05 vs. HG. See Supplementary Figure 5 for uncropped scans of the western blot.

Our previous study showed that Sirt1 activated the Nrf2/ARE pathway in HG-induced GMCs (Huang et al., 2013). For further exploring the way of Nrf2 activation after PA treatment, expression and nuclear accumulation of Sirt1 was examined in HG-treated GMCs. As shown in **Figure 5A**, HG treatment for 6 h had no obvious effect on the expression of total Sirt1, whereas PA slightly up-regulated total Sirt1 level. Further study with nuclear extract showed that PA obviously promoted Sirt1 nuclear translocation in GMCs induced by HG 6 h (**Figure 5B**). Additionally, HG treatment for 24 h decreased total and nuclear Sirt1 levels, which were both reversed by PA treatment (**Figures 5C,D**).

### Sirt1 Mediated the Effects of PA on the Nrf2 Pathway in GMCs Treated With Short-Time HG

**FIGURE 6:**
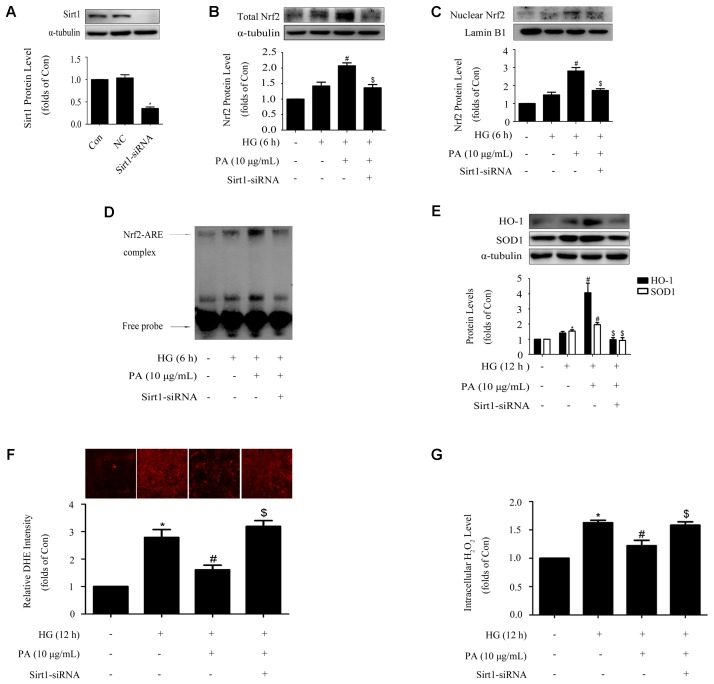
Sirt1 mediated the effects of PA on the Nrf2 pathway in GMCs treated with short-time HG. **(A)** Confirmation of silencing efficiency of Sirt1-siRNA. GMCs were subjected to Sirt1 knockdown with siRNA for 48 h. Then, the protein extracts were subjected to immunoblot analysis with the antibody against Sirt1. **(B)** Nuclear Nrf2 expression under Sirt1 knockdown conditions. **(C)** EMSA for measurement of Nrf2/ARE binding activity. **(D)** Western blot analysis of Nrf2 under Sirt1 knockdown conditions. **(E)** HO-1 and SOD1 protein levels were measured by immunoblot analysis under Sirt1 knockdown conditions. **(F)** Analysis of DHE fluorescence intensity by high content screening system. **(G)** Quantification of H_2_O_2_. The above experiments were performed at least three times with similar results. ^∗^*P* < 0.05 vs. Con, ^#^*P* < 0.05 vs. HG, ^$^*P* < 0.05 vs. HG + PA. See Supplementary Figure 6 for uncropped scans of the western blot.

Protein level of Sirt1 was efficiently knocked down by the method of siRNA, as confirmed by WB (**Figure 6A**). Knockdown of Sirt1 not only suppressed PA-induced nuclear translocation of Nrf2 and activation of ARE binding activity (**Figures 6B,C**), but also declined the up-regulation of total Nrf2 during PA treatment (**Figure 6D**), which was accompanied by decreases of HO-1 and SOD1 levels (**Figure 6E**) and increases of superoxide and H_2_O_2_ generations in GMCs (**Figures 6F,G**).

### Sirt1 Mediated the Effects of PA on the Nrf2 Pathway in GMCs Treated With Long-Time HG

**FIGURE 7:**
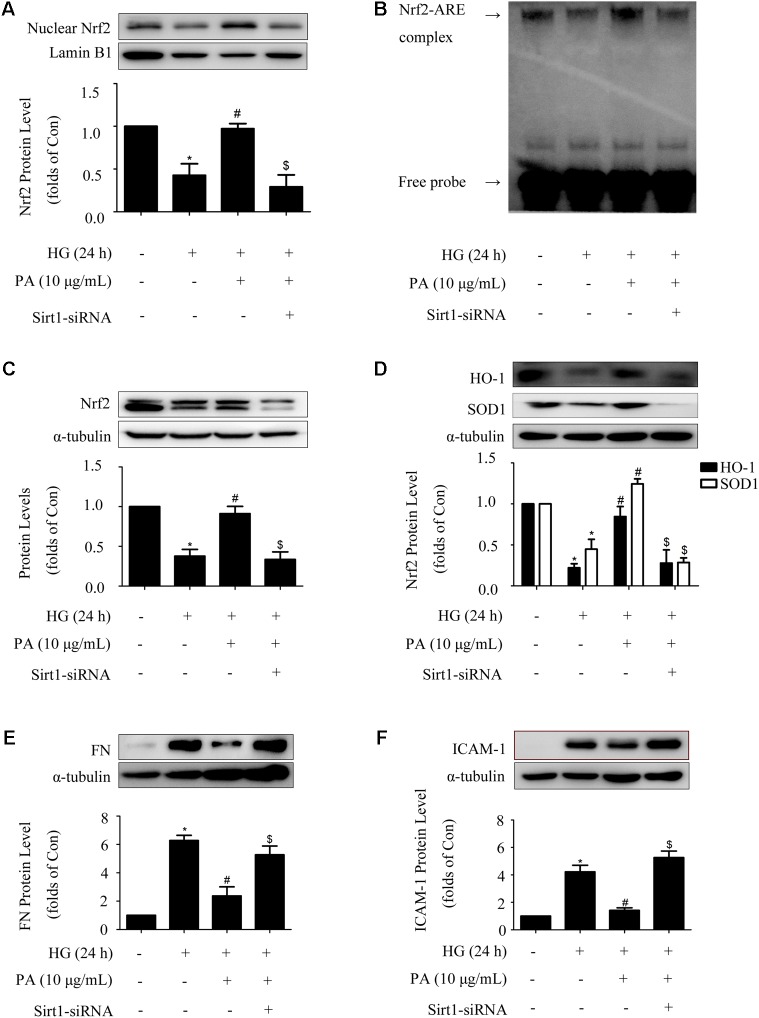
Sirt1 mediated the effects of PA on the Nrf2 pathway in GMCs treated with long-time HG. **(A)** Immunoblot images of nuclear Nrf2 in GMCs under Sirt1 knockdown conditions. **(B)** EMSA for measurement of Nrf2/ARE binding activity under Sirt1 knockdown conditions. **(C)** Western blot analysis of Nrf2 using α-tubulin as a loading control. **(D)** Immunoblot images and quantitative representations of HO-1 and SOD1 in GMCs. **(E,F)** Immunoblot images and quantitative representations of FN and ICAM-1 in GMCs. The above experiments were performed at least three times with similar results. ^∗^*P* < 0.05 vs. Con, ^#^*P* < 0.05 vs. HG, ^$^*P* < 0.05 vs. HG + PA. See Supplementary Figure 7 for uncropped scans of the western blot.

Sirt1-siRNA reversed the effects of PA on the Nrf2 pathway in GMCs treated with short-time HG, whether Sirt1-siRNA did the same in GMCs treated with long-time HG remained unclear. Our results showed that knockdown of Sirt1 reversed PA-induced Nrf2 nuclear accumulation and activation of ARE binding activity in long-time HG-treated GMCs (**Figures 7A,B**). Moreover, up-regulations of total Nrf2 and antioxidant enzyme (i.e., HO-1 and SOD1) expressions during PA treatment were also reversed by Sirt1-siRNA (**Figures 7C,D**). Last but not least, Sirt1-siRNA abrogated the inhibitory effects of PA on expressions of FN and ICAM-1 in long-time HG treated GMCs (**Figures 7E,F**). These results indicated that Sirt1 was involved in the regulation of PA on the Nrf2 pathway in GMCs.

### PA Ameliorated Renal Function and Renal Fibrosis in STZ-Induced Diabetic Mice

The above experiments *in vitro* demonstrated that PA exerted regulatory effects on Nrf2 and Sirt1 as well as inflammatory fibrotic factors such as FN and ICAM-1 in HG-induced GMCs. However, further work *in vivo* is needed to elucidate the essential role of PA in diabetic mice. Data showed diabetic mice exhibited prominent renal function injuries compared with the normal controls. As shown in **Table 1**, FBG levels were elevated in the model group compared with the normal controls. Administration of PA reduced FBG levels, almost comparable to metformin, a first-line medication for the treatment of type 2 diabetes. In addition, KW/BW, BUN, Cr, and 24 h UP were dramatically increased in the model group, whereas PA declined KW/BW, BUN, Cr, and 24 h UP of diabetic mice after 8-week treatment.

**Table 1:** The renal function parameters of diabetic mice after PA treatment.

Parameters (*n* = 8)	Control (*n* = 8)	Diabetes (*n* = 8)	PA (*n* = 8)	ME (*n* = 8)
FBG (mmol/L)	5.86 ± 0.42	18.26 ± 1.24*	9.32 ± 1.61#	6.05 ± 0.91#
Body weight (g)	29.71 ± 0.78	22.44 ± 0.29*	21.78 ± 0.29	22.53 ± 0.36
KW/BW (%)	1.19 ± 0.30	1.53 ± 0.02*	1.19 ± 0.03#	1.38 ± 0.05#
BUN (mmol/L)	11.61 ± 0.19	22.66 ± 0.75*	13.02 ± 0.68#	13.18 ± 0.74#
Cr (μmol/L)	13.60 ± 2.06	21.13 ± 1.09*	10.39 ± 0.71#	15.73 ± 0.65#
UP 24 h (mg/24 h)	2.27 ± 0.25	7.14 ± 0.14*	3.32 ± 0.50#	1.52 ± 0.27#

*Each value represents mean ± SEM. FBG, fasting blood glucose; KW/BW, kidney/body weight ratio; BUN, blood urea nitrogen; Cr, serum creatinine; UP 24 h, urine protein for 24 h; PA, paeonol; ME, metformin. ^∗^P < 0.05 vs. Con, ^#^P < 0.05 vs. Diabetes*.

**FIGURE 8:**
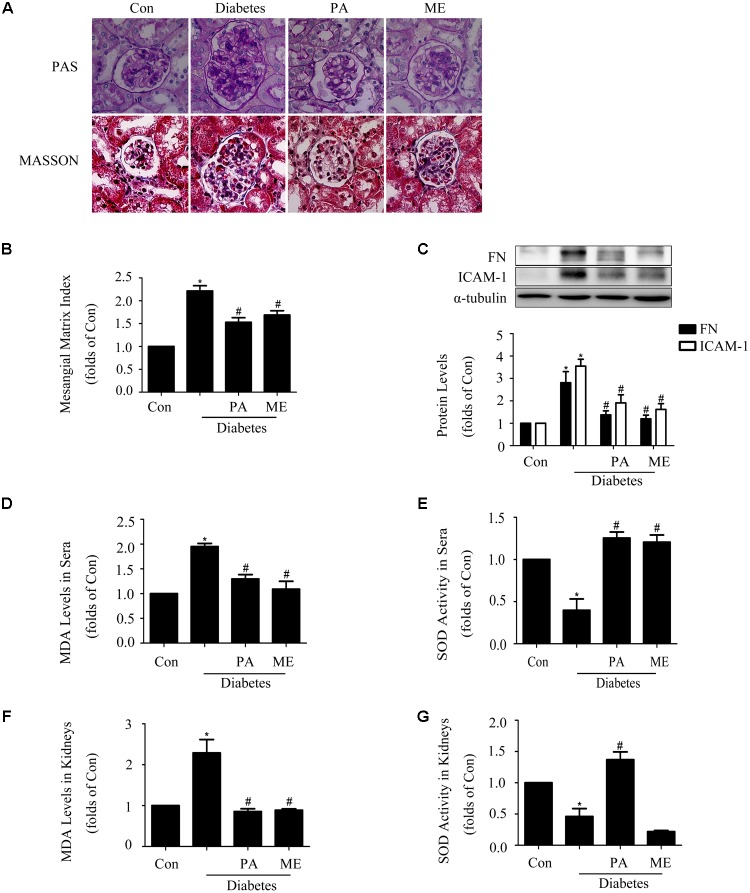
Paeonol ameliorated renal function and renal fibrosis in STZ-induced diabetic mice (*n* = 4–6). **(A,B)** Pathological changes of glomerular in diabetic mice, assessed by PAS and MASSON staining (200-fold magnification). **(C)** Immunoblot assessments of FN and ICAM-1 in diabetic kidneys. **(D,E)** Measurements of MDA levels and SOD activity in sera of diabetic mice. **(F,G)** Measurements of MDA levels and SOD activity in kidneys. The above experiments were performed at least three times with similar results. ^∗^*P* < 0.05 vs. Con, ^#^*P* < 0.05 vs. Diabetes. See Supplementary Figure 8 for uncropped scans of the western blot.

We further observed the effects of PA on glomerular lesions of diabetic mice. The elevated mesangial expansion, basement membrane thickening, glomerular adhesion and collagen deposition seen in diabetic mice were improved by PA or ME treatment (**Figure 8A**). What is more, PA treatment suppressed mesangial matrix expansion in kidneys, while there was a significant increase (twofold) in mesangial matrix expansion in diabetic mice compared with non-diabetic control mice (**Figure 8B**).

Fibronectin and ICAM-1 expression levels were significantly up-regulated in the kidney tissues of diabetic mice. Moreover, these up-regulations were inhibited by PA or ME treatment (**Figure 8C**). We then examined whether PA improved anti-oxidative capacity of kidneys. MDA level in sera and kidneys were increased in the model group, which was significantly suppressed by PA or ME treatment (**Figures 8D,F**). SOD activity in sera and kidneys were decreased in the diabetic mice, whereas these reductions were improved when mice were treated with PA (**Figures 8E,G**). In this study, we detected the elevated effects of ME on SOD activity in sera but not in kidneys of diabetic mice (**Figures 8E,G**).

### PA Elevated Sirt1 and Nrf2 Expressions in the Kidneys of Diabetic Mice

**FIGURE 9:**
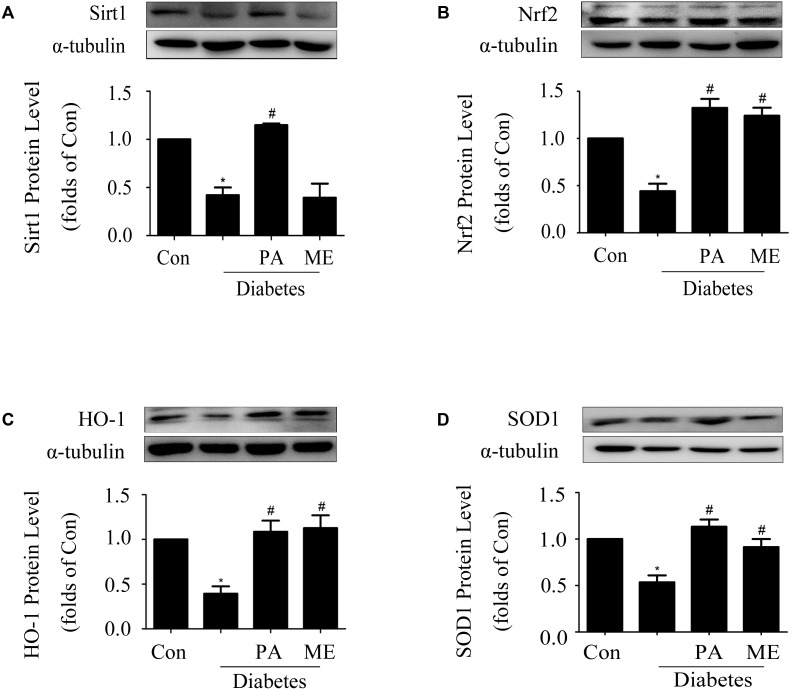
Paeonol elevated Sirt1 and Nrf2 expressions in the kidneys of diabetic mice (*n* = 4–6). **(A,B)** Immunoblot analysis of Sirt1 and Nrf2 in diabetic kidneys. Representative immunoblots with matching α-tubulin controls in bottom panel. **(C,D)** Protein expressions of HO-1 and SOD1 assessed in diabetic kidneys. The above experiments were performed at least four times with similar results. ^∗^*P* < 0.05 vs. Con, ^#^*P* < 0.05 vs. Diabetes. See Supplementary Figure 9 for uncropped scans of the western blot.

Finally, we assessed the effects of PA on Sirt1 and the Nrf2 pathway in diabetic mice. PA enhanced Sirt1, Nrf2, HO-1, and SOD1 expressions in the kidneys of diabetic mice (**Figures 9A–D**), which was consistent with the *in vitro* results, while metformin enhanced Nrf2, HO-1, and SOD1 expression (**Figures 9B–D**) excluding Sirt1 (**Figure 9A**). Taken together, these *in vitro* and *in vivo* results confirmed that PA inhibited development of diabetic renal fibrosis at least partially via activating the Nrf2/ARE pathway in the HG microenvironment, leading to reductions in expressions of FN and ICAM-1 in GMCs and thus inhibiting DN.

## Discussion

Cortex Moutan is a herb commonly found in the traditional Chinese formula, such as Liuwei Dihuang Wan, for the treatments of diabetes (Hui, 2000). PA has been identified as one of the active compounds with anti-diabetic activity extracted from Cortex Moutan by previous study (Lau et al., 2007). Moreover, PA also possesses a large variety of pharmacological activities, such as anti-tumor, anti-hypertension, and anti-oxidation (Sun et al., 2008; Chen et al., 2012; Zhang et al., 2013; Gong et al., 2017). PA was reported to exhibit significant hydroxyl radical scavenging activities (Jin et al., 2016) and inhibit the levels of iNOS and nitric oxide in LPS/D-GalN-induced acute liver failure models (Gong et al., 2017), implying strong anti-oxidant actions of PA. As oxidative stress makes great contributions to DN, we speculated whether PA could improve DN through its anti-oxidant effects.

Recent studies demonstrated that PA reduces blood glucose levels and improves the pathological damage of diabetic encephalopathy in STZ-induced diabetic rats by modulating AGEs/RAGE/NF-κB pathway (Liu et al., 2013). Additionally, PA reduces ROS production and decreases FN and TGF-β1 levels in HG-treated GMCs. All these reports indicated a possible anti-fibrotic effect of PA and that effect might be correlated with its anti-oxidative actions. Interestingly, several studies revealed that other herbal products up-regulates Nrf2 and HO-1 and thus ameliorates trimethyltin-induced neuronal damage (Catino et al., 2015), improves cognitive skills (Mhillaj et al., 2018), and protects against noise-induced injury in rat cochlea (Fetoni et al., 2015). We and others showed an efficient regulation of Nrf2 and HO-1 activation induced by herbal products on various diseases, which provides experimental evidence for antioxidant therapy for various diseases. In the present study, the anti-fibrotic effect of PA was confirmed by reductions of FN and ICAM-1 expression levels *in vitro* and *in vivo*. And the anti-oxidative effect of PA was verified by down-regulations of oxidative stress-related markers in both HG-induced GMCs and diabetic kidneys.

Several published studies have shown Nrf2 is adaptively trying to remain functional to overcome diabetic damage at the early stage of diabetes, but showed an unavoidable decrease attributed to impaired antioxidant function at the late stage of diabetes (He et al., 2009; Tan et al., 2011). Our previous studies have also verified that Nrf2 as an adaptive mechanism is activated in HG-induced GMCs (Gong et al., 2016). Thus, we examined the protective effects of PA in both short- and long-time HG treated GMCs. The results demonstrated that PA up-regulated the nuclear content of Nrf2 and promoted transcription of its downstream antioxidant genes under both short-time and long-time HG treatment conditions, thus quenching ROS overproduction and FN and ICAM-1 accumulations in GMCs. Moreover, Nrf2 was required for PA to prevent up-regulations of ROS and inflammatory fibrotic factors. After Nrf2 depletion, the inhibitory effects of PA on ROS, FN, and ICAM-1 were overridden. Our *in vivo* results showed a significant decrease in expressions of HO-1 and SOD1 in diabetic kidneys, which indicated an oxidative stress injury. However, HO-1 was demonstrated to be up-regulated in the lymphocytes of subjects with DN. This difference may be due to the immune system is trying to counteract the oxidant insult by inducing early genes (Calabrese et al., 2007). The HO-1 reduction observed in the kidneys of diabetic mice in this study suggests an unavoidable impairment in antioxidant system, which provides interesting insight into the DN field.

Sirt1 is a promising target for chronic kidney diseases, including DN (Kume et al., 2013). Sirt1 was found to inhibit the activation of inflammatory signals, ROS-mediated apoptosis and HG-induced hypertrophy in various types of renal cells (Yeung et al., 2004; Kume et al., 2006; Zhuo et al., 2011). Sirt1-deficient mice develop premature renal aging, worsening fibrosis, increased proteinuria and renal function impairment (He et al., 2010; Kume et al., 2010; Liu et al., 2014). Our previous study has also verified the involvement of Sirt1 in DN (Huang et al., 2013). In the present study, we found that PA promoted Sirt1 expression *in vitro* and *in vivo*. Furthermore, Sirt1 was required for PA to activate the Nrf2-pathway. However, to the best of our knowledge, this was the first report to reveal the mechanism of PA for its anti-DN effects.

Our previous studies showed that Sirt1 can promote Nrf2 nuclear aggregation, DNA binding activity and transcriptional activity, as well as upregulate the expression of Nrf2 downstream target genes (Huang et al., 2013). In addition, studies have reported that Sirt1 stabilizes Nrf2 protein expression by deacetylating Nrf2 to reduce its ubiquitination level (Ding et al., 2016). Zhou et al. (2015) showed that Sirt1 reduced the expression of miR-29 and reduced the expression of Keap1 by deacetylating p65, which facilitated the translocation of Nrf2 into the nucleus. Our results show that PA can increase Sirt1 protein expression and nuclear content, thereby increasing the nuclear level of Nrf2. Therefore, we speculated that PA might increase the total protein expression and nuclear content of Nrf2, and activate the expression of downstream target genes, by up-regulating Sirt1 protein expression and reducing the acetylation level of Nrf2, eventually leading to inhibition of the ubiquitination and degradation of Nrf2.

Metformin was chosen as the positive control in animal trials to evaluate the anti-diabetic effects of PA. Metformin had been widely used in patients with type 2 diabetes mellitus diabetes. In this study, we observed marked reductions of blood glucose levels in PA-administrated group after 8-week treatments, almost compared to metformin group, indicating the good anti-diabetic effect of PA. And we hypothesized the mechanism was likely through inhibiting intestinal glucose absorption based on previous study (Lau et al., 2007).

During the experiment periods, PA showed no obvious adverse effects on mice. Previous study found that intragastric LD_50_ value of PA in mice is 3430 mg/kg body wt., which indicated that the toxicity of PA is relatively low (Harada and Yamashita, 1969). Indeed, PA has been used in the treatment of fever, headache and dermatitis-eczema as the tablet formulation. Our study provides new insights into the anti-oxidative mechanism of PA on resisting DN and the application of PA in DN treatment. However, the poor water solubility of PA limited its wide use in clinical applications. More researches are needed to resolve this problem and develop more new anti-DN drugs with high efficacy based on the template of PA.

## Conclusion

Our study shows that PA significantly reduces ROS overproduction and eventually inhibits the expressions of FN and ICAM-1 in both HG-induced GMCs and STZ-induced diabetic mice. The underlying mechanism is through up-regulating Sirt1 expression to activate the Nrf2 pathway, resisting oxidative stress in DN. These data suggest that PA could suppress chronic diabetic renal fibrosis thus slowing the progression of DN. Our data provide preliminarily experimental evidence for the anti-oxidant role of PA in the prevention of DN diseases, and further investigation will be needed to explore if PA is a significant target for treatment in DN.

## Ethics Statement

Male C57/BL6 mice were purchased from Laboratory Animal Center, Sun Yat-sen University, Guangzhou, China (Animal Quality Certificate Number: 44007200039420). All the procedures were done in compliance with the China Animal Welfare Legislation and were reviewed and approved by the Sun Yat-sen University Committee on Ethics in the Care and Use of Laboratory.

## Author Contributions

LZ: contributed to study design, data acquisition, manuscript preparation, and editing. WG: contributed to data acquisition, analysis, and interpretation. ZC: contributed to study concepts and interpretation of data. YZ: contributed to study concepts and manuscript editing. FX and LC: contributed to manuscript preparation and statistical analysis. HH: contributed to the design of the work and manuscript revision/review.

## Conflict of Interest Statement

The authors declare that the research was conducted in the absence of any commercial or financial relationships that could be construed as a potential conflict of interest.

## Abbreviations

ARE: antioxidant response element
BUN: blood urea nitrogen
Cr: serum creatinine
DN: diabetic nephropathy
ECM: extracellular matrix
FBG: fasting blood glucose
FN: fibronectin
GBM: glomerular basement membrane
GMCs: glomerular mesangial cells
H_2_O_2_: hydrogen peroxide
HG: high glucose
HO-1: heme oxygenase 1
ICAM-1: intercellular adhesion molecule-1
KW/BW: kidney/body weight ratio
MDA: malondialdehyde
Nrf2: nuclear factor E2-related factor 2
PA: paeonol
ROS: reactive oxygen species
Sirt1: silent information regulator 2-related protein 1
SOD: superoxide dismutase
STZ: streptozocin
TGF-β1: transforming growth factor-β1
UP: urine protein
