# Supplementary material for: Paeonol Ameliorates Diabetic Renal Fibrosis Through Promoting the Activation of the Nrf2/ARE Pathway via Up-Regulating Sirt1
Source: Front Pharmacol. 2018 May 18;9:512. doi: 10.3389/fphar.2018.00512 (PMC5968333; doi:10.3389/fphar.2018.00512)

Supplementary original data: Uncropped western blots for Figure 1.

Fig 1D Western blot anti FN and ICAM-1

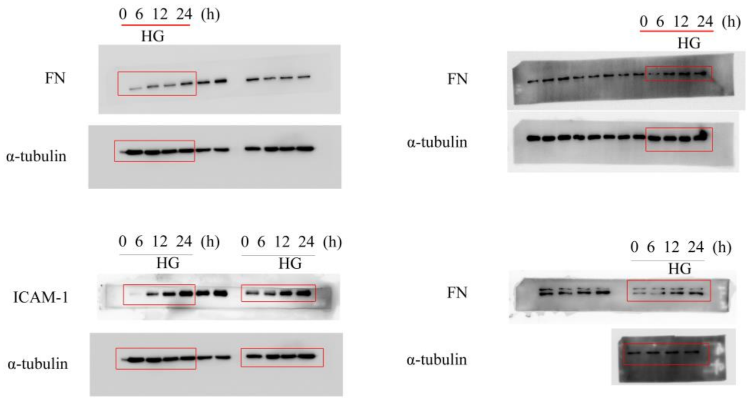

Fig 1E Western blot anti FN

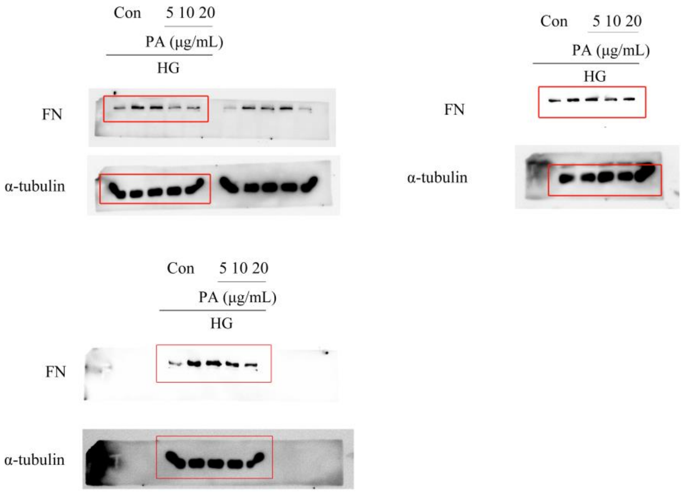

Fig 1E Western blot anti ICAM-1

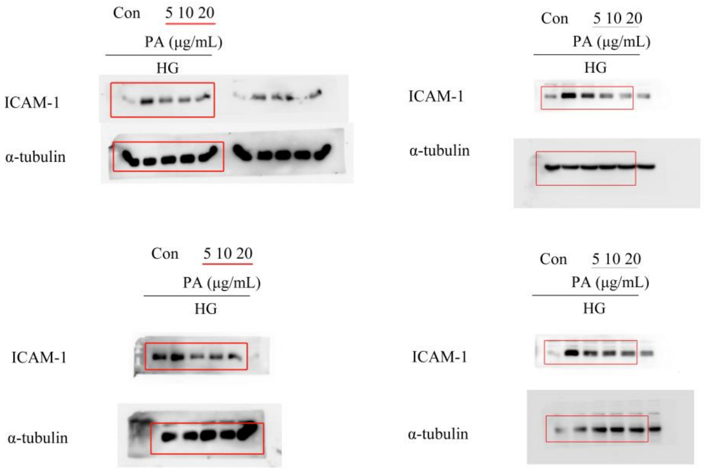

Supplementary original data: Uncropped western blots for Figure 2.

Note:

Con	HG	HG+5	HG+10	HG+20
1	2	3	4	5

Fig 2A Western blot anti Nrf2

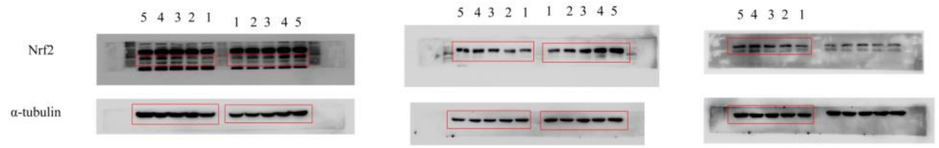

Fig 2B Western blot anti Nrf2

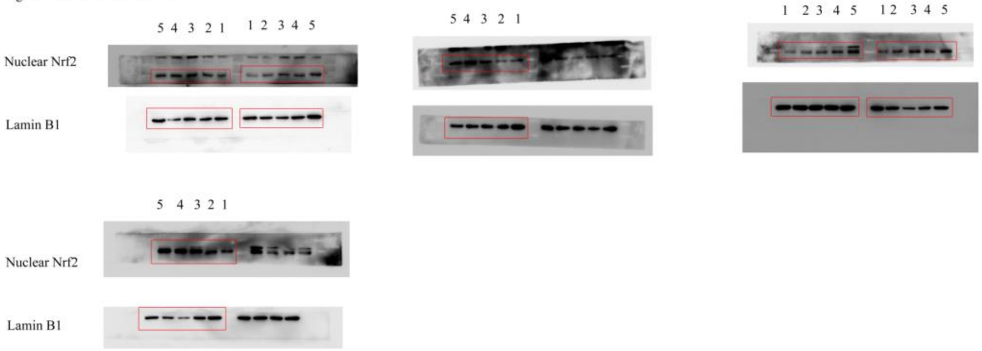

Fig 2D Western blot anti HO-1

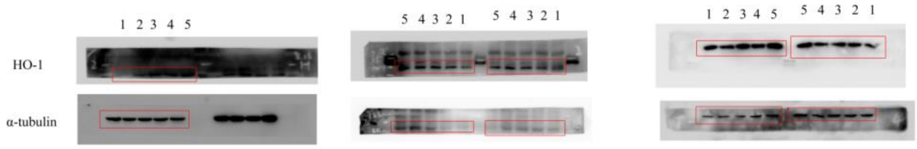

Fig 2E Western blot anti SOD1

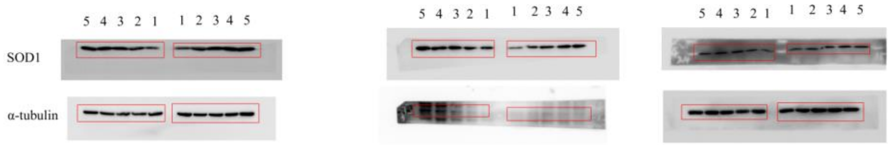

Supplementary original data: Uncropped western blots for Figure 3.

Note:

Con	HG	HG+5	HG+10	HG+20
1	2	3	4	5

Fig 3A Western blot anti Nrf2

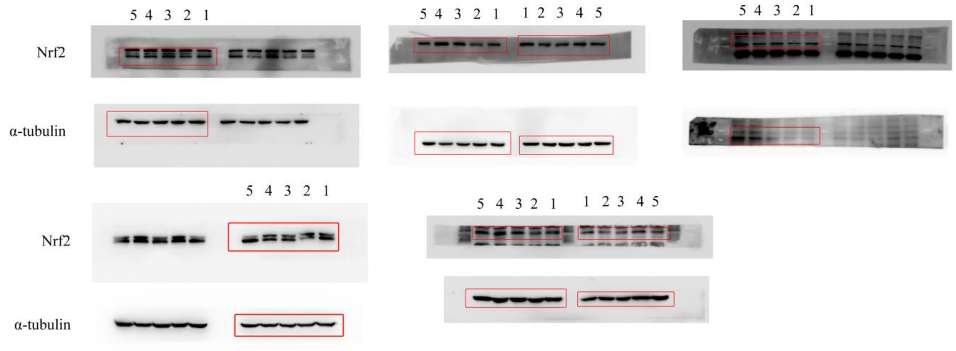

Fig 3B Western blot anti Nrf2

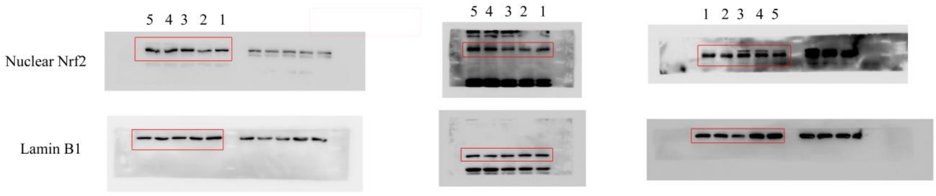

Fig 3C Western blot anti HO-1

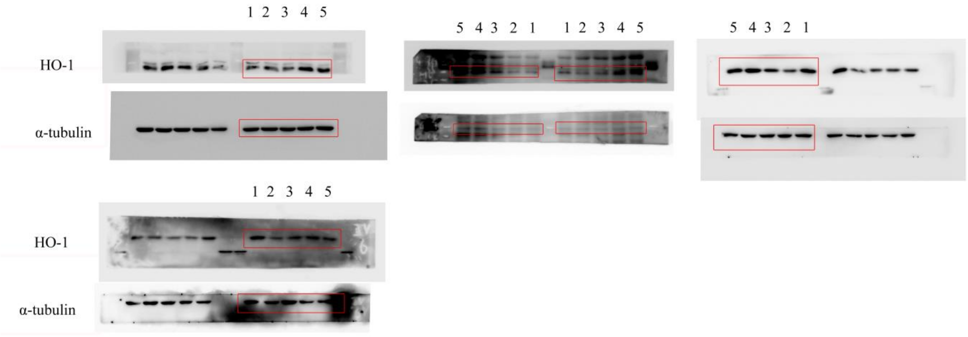

Fig 3D Western blot anti SOD1

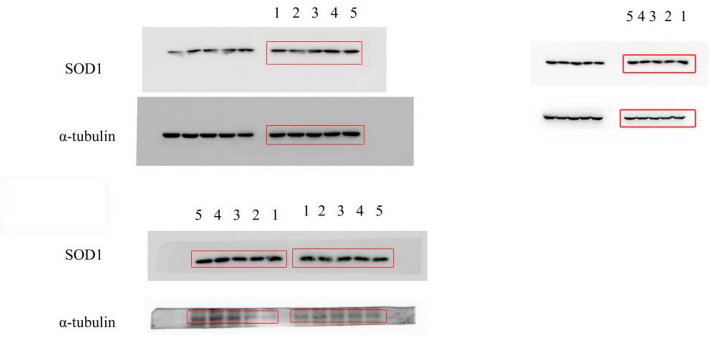

Supplementary original data: Uncropped western blots for Figure 4.

Note: Con, HG, HG+PA, HG+PA+Si
1 2 3 4

Fig 4A Western blot anti Nrf2

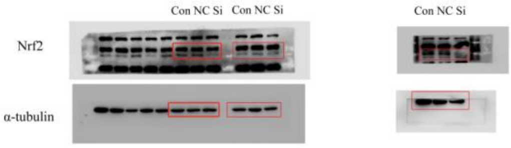

Fig 4B Western blot anti HO-1

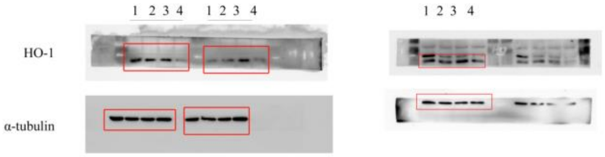

Fig 4C Western blot anti SOD1

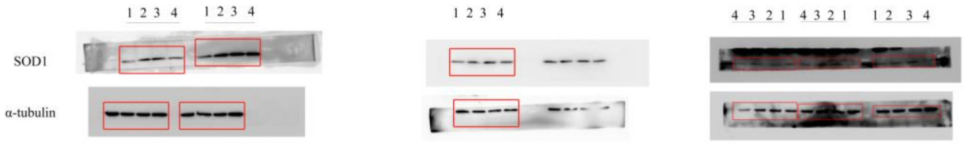

Fig 4F Western blot anti HO-1

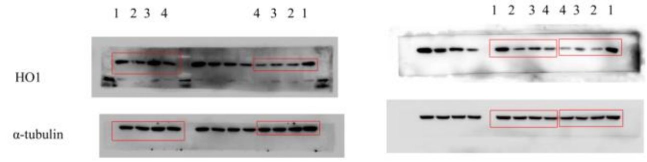

Fig 4G Western blot anti SOD1

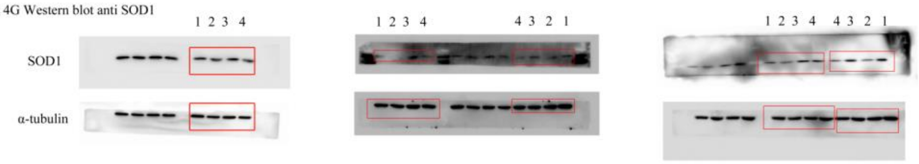

Fig 4H Western blot anti FN

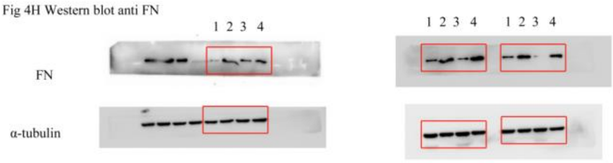

Fig 4I Western blot anti ICAM-1

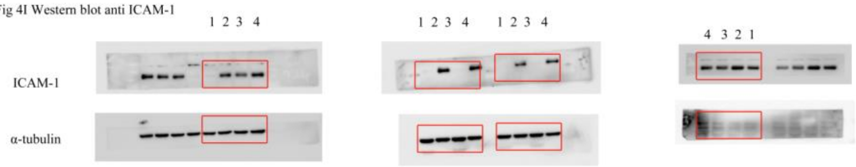

Supplementary original data: Uncropped western blots for Figure 5.

Note:

Con	HG	HG+5	HG+10	HG+20
1	2	3	4	5

Fig 5A Western blot anti Sirt1

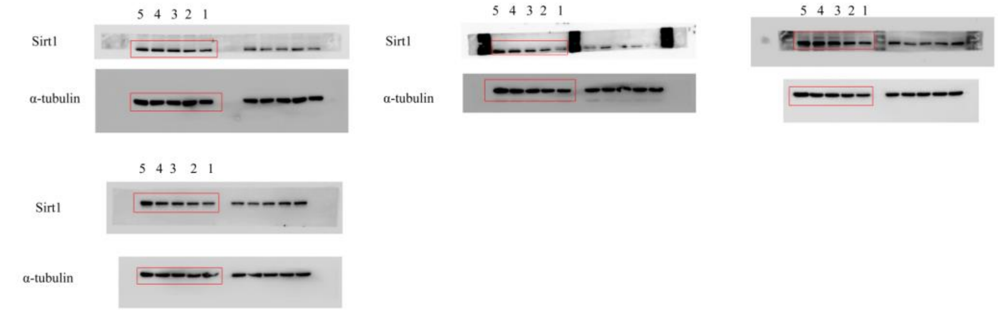

Fig 5B Western blot anti Sirt1

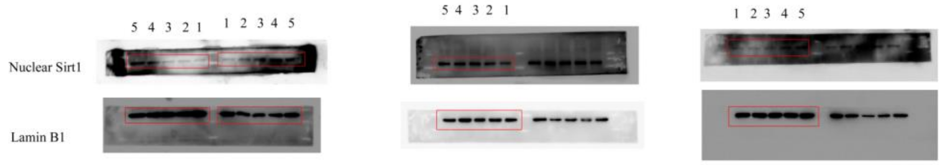

Fig 5C Western blot anti Sirt1

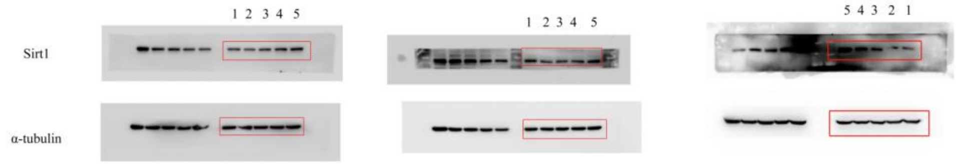

Fig 5D Western blot anti Sirt1

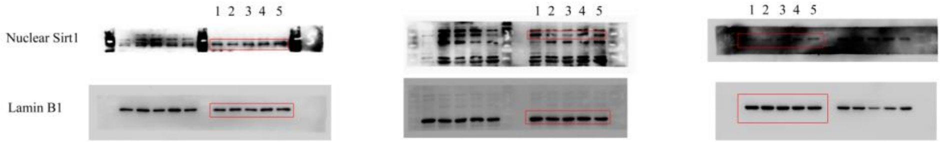

Supplementary original data: Uncropped western blots for Figure 6.

Fig 6A Western blot anti Sirt1

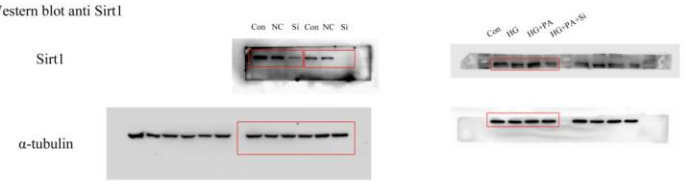

Fig 6B Western blot anti Nrf2

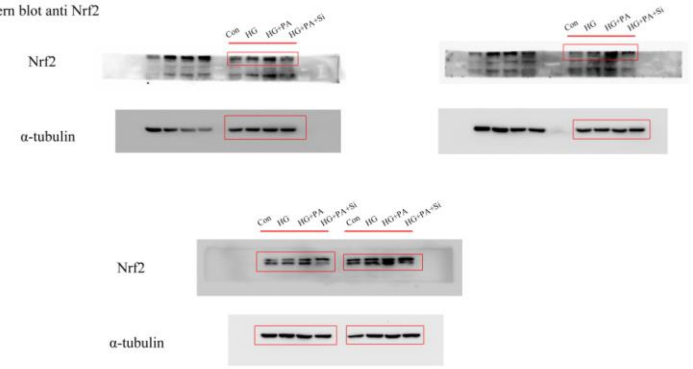

Fig 6C Western blot anti Nrf2

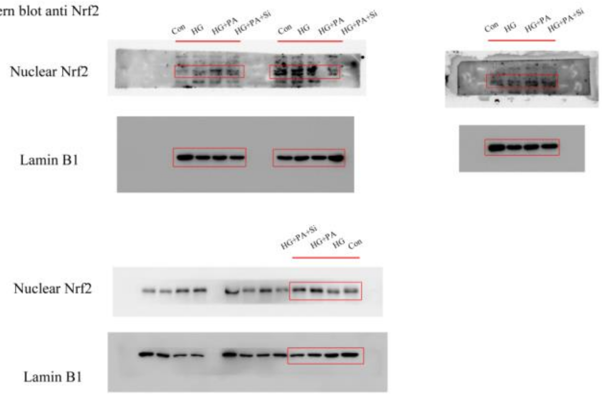

Fig 6E Western blot anti HO-1

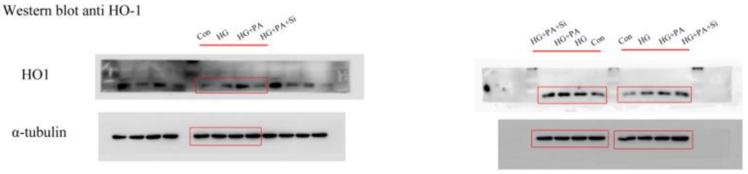

Fig 6E Western blot anti SOD1

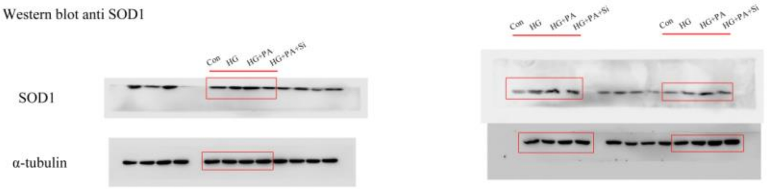

Supplementary original data: Uncropped western blots for Figure 7.

Note: Con HG HG+PA HG+PA+Si
1 2 3 4

Fig 7A Western blot anti Nrf2

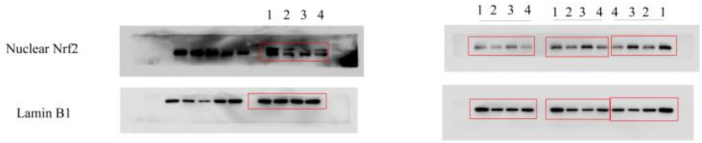

Fig 7C Western blot anti Nrf2

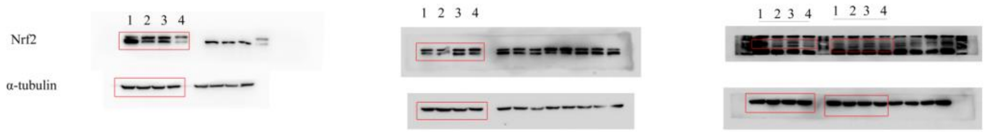

Fig 7D Western blot anti HO-1

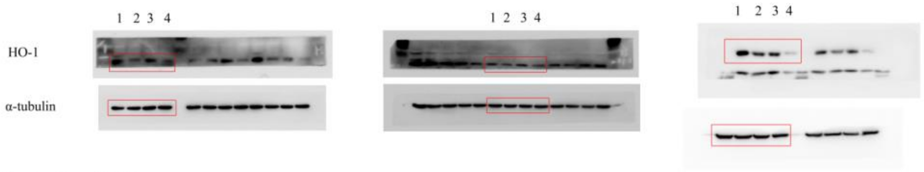

Fig 7D Western blot anti SOD1

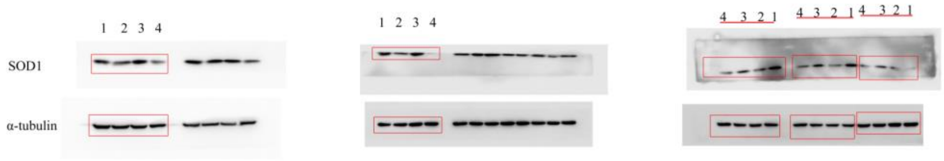

Fig. 7E Western blot anti FN

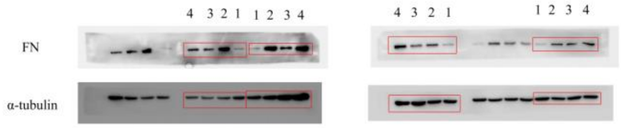

Fig. 7F Western blot anti ICAM-1

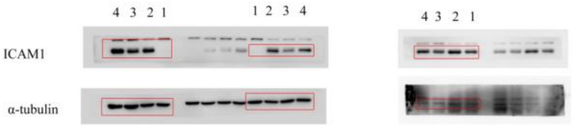

Supplementary original data: Uncropped western blots for Figure 8.

Note: Con Diabetes PA ME
1 2 3 4

Fig 8C Western blot anti FN and ICAM-1

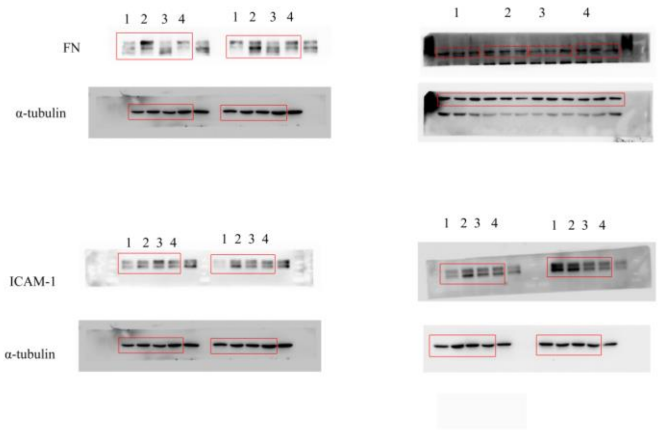

Supplementary original data: Uncropped western blots for Figure 9.

Note: Con Diabetes PA ME
1 2 3 4

Fig 9A Western blot anti Sirt1

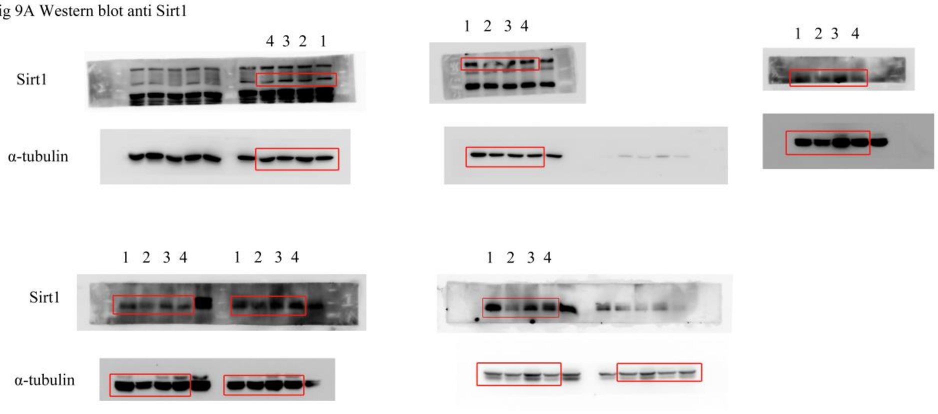

Fig 9B Western blot anti Nrf2

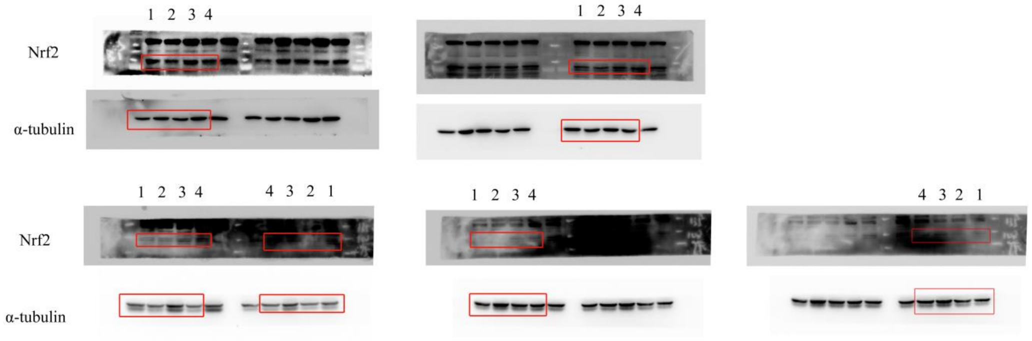

Fig 9C Western blot anti HO-1

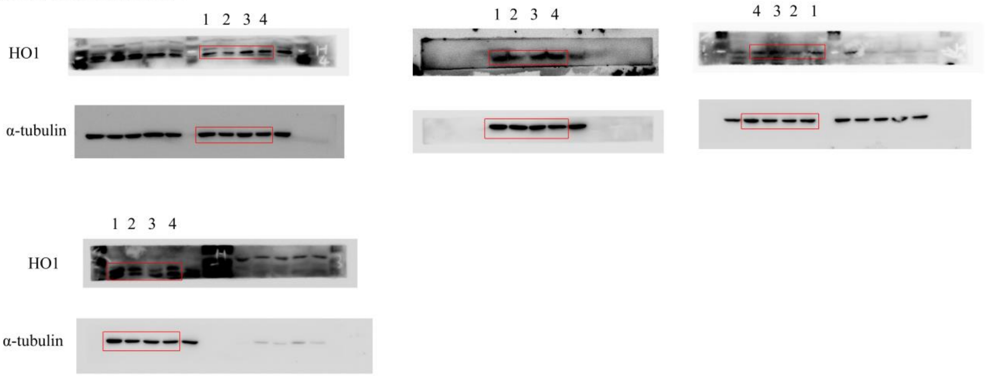

Fig 9D Western blot anti SOD1

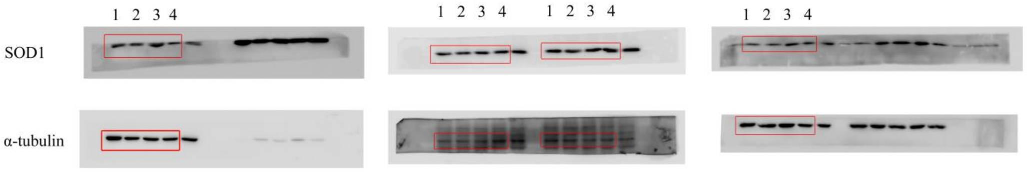

Supplement: Supplementary file 1 [file Presentation_1.pdf]
